# The Anatomical and Functional Heterogeneity of the Mediodorsal Thalamus

**DOI:** 10.3390/brainsci10090624

**Published:** 2020-09-09

**Authors:** Ioana Antoaneta Georgescu, Daniela Popa, Leon Zagrean

**Affiliations:** 1 Division of Physiology and Neuroscience, Carol Davila University of Medicine and Pharmacy, Eroii Sanitari, nr 8, Sector 5, 050474 Bucharest, Romania; antoaneta.georgescu@drd.umfcd.ro; 2 Institut de biologie de l’Ecole normale supérieure (IBENS), Ecole Normale Supérieure, CNRS, INSERM, PSL Research University, 75005 Paris, France

**Keywords:** mediodorsal thalamus, limbic system, prefrontal cortex, anatomy, cognition, emotion, behavior

## Abstract

The mediodorsal nucleus (MD) represents just one piece of a complex relay structure situated within the brain, called the thalamus. MD is characterized by its robust interconnections with other brain areas, especially with limbic-related structures. Given the close anatomo-functional relationship between the MD and the limbic system, this particular thalamic nucleus can directly influence various affective behaviors and participate in cognition. In this work, we review data collected from multiple anatomical studies conducted in rodent, human, and non-human primates, highlighting the complexity of this structure and of the neural networks in which it takes part. We provide proof that the MD is involved in the unification of several anatomical structures, being able to process the information and influence the activity in numerous cortical and subcortical neural circuits. Moreover, we uncover intrinsic and extrinsic mechanisms that offer MD the possibility to execute and control specific high functions of the nervous system. The collected data indicate the great importance of the MD in the limbic system and offer relevant insight into the organization of thalamic circuits that support MD functions.

## 1. Introduction

The thalamus has long been known as an essential sensory relay station that filters information and acts as a gate between the body and the brain. Nevertheless, the role of the thalamus is highly complex and the limbic processes for which it is responsible have been considered only to a small extent [1]. The mediodorsal nucleus is part of the limbic thalamus, as it is closely linked to multiple structures belonging to the limbic system. The prefrontal cortex, amygdala, basal ganglia, spinothalamic fibers and olfactory cortex represent the main afferents of the midline group of the thalamic nuclei, while the medial and lateral prefrontal cortices and the orbitofrontal cortex stand for the efferents [2]. The MD carries out its activity mainly in a circuit held within the basolateral amygdala and prefrontal cortex (PFC) and can disconnect the cortex from the medial temporal lobes [3]. Damaging the MD can cause disconnection syndromes that are mediated by the frontal lobes. This fact will negatively affect executive functions, including behavioral flexibility, strategy shifting, and reversal learning [4]. The MD is critically involved in cognition, given the bidirectional connectivity and partnership that it creates with the prefrontal cortex. The working memory, attention control, planning, decision making, reward evaluation, sensory discriminations, learning, and other different forms of memory processing have all been proven to require MD participation [5,6,7].

The cellular morphology helped differentiate the MD subdivisions [8] and identify specific routes formed by them. However, even though it is recognized that MD supports cognitive and affective activities, the way the neural circuits communicate is still unclear. In addition, a certain reevaluation of how functions are attributed to specific MD regions and pathways must be done. Our research collects and organizes the existing data and shapes the anatomy of several cortico-subcortical circuits MD is part of. This review offers new insight towards the physiological anatomy of the MD and underlies important neural mechanisms that allow MD to exert its role in the brain.

## 2. Thalamus Architecture

Thalamus is a bilateral midline structure with two symmetrical halves attached by massa intermedia (the interthalamic adhesion). In humans, it is located just above the brainstem, between the left and right lateral ventricles and the third ventricle so that the medial surface of the thalamus touches the top lateral wall of the third ventricle [2,9]. Thalamus represents a diencephalic gray matter structure that works as an integrative center: Its damage may cause sensory loss (tactile sensation and discrimination, auditory and visual disturbances), burning or freezing sensations in addition to intense pain, seizures, insomnia, memory loss, depression, and executive functions deficits [10]. It is an egg-shaped structure, covered by two layers of white matter: stratum zonale (on the superior surface) and external medullary lamina (on the lateral sides). The internal medullary lamina (an upright Y-shaped sheet bundle of myelinated fibers) splits the thalamic gray matter into three segments: anterior (in the space separated by the two arms of the Y), medial, and lateral. In the anterior part, we can find the anterior nuclear complex, represented by the anterodorsal, anteromedial, and anteroventral nuclei. They receive input from the hippocampus and subiculum via the fornix (directly) and mammilothalamic tract (indirectly) and send efferent fibers to the cingulate gyrus, limbic, and orbitofrontal cortex. The lateral part of the thalamus has two tiers of nuclei: a dorsal (containing the lateral dorsal nucleus, the lateral posterior nucleus and the pulvinar) and a ventral one (containing the ventral anterior, ventral lateral, ventral medial, ventral posterior, postero-lateral, postero-medial, and postero-inferior nuclei). The lateral nuclei are part of the somatosensory system. In monkeys, they receive input from the cerebellum and basal ganglia and project to the premotor cortex, supplementary, caudal and rostral premotor areas, cingulate [11], and even primary vestibular cortices [12]. The reticular nucleus of the thalamus, consisting of a thin neuronal wall, covers entirely the lateral side of the human thalamus, which contains nuclei located outside of the internal medullary lamina.

The medial region of the human thalamus includes the mediodorsal nucleus (MD) and some midline structures, represented by reuniens, rhomboid, paraventricular, and paratenial nuclei. The nucleus reuniens has a bidirectional relationship with the prefrontal cortex and sends output to the entorhinal cortex. Midline and intralaminar nuclei receive substantial input from the brainstem and are involved in multiple functions (including cognitive, motor, and sensory). Therefore, they might play a role in awareness and arousal of multiple cortical regions. Moreover, they are considered part of the ascending reticular activating system. The intralaminar group contains the centrolateral, centromedial, paracentral, and parafascicular nuclei. The metathalamus, found at the posterior part of pulvinar, holds two relay structures: The medial geniculate nucleus (for hearing) and the lateral geniculate nucleus (for vision) [13,14]. This brief description of the thalamus may seem complicated, but interestingly, the researchers discovered more than 40 distinct nuclei in the human thalamus, all associated with various functional systems [15].

The mammalian thalamus has a similar nuclear arrangement and connectivity. The elementary structure and the cytoarchitecture of the rodent, cat, and monkey thalami resemble each other [16,17] and are comparable to the human one, summarized above [13]. However, the exact location of the thalamus in the mouse brain is different than in humans: It is situated anterior to the brainstem and rostral to the midbrain [18].

## 3. Mediodorsal Thalamic Relationship with the Prefrontal Cortex

Higher-order thalamic nuclei are vitally involved in cortical functions by providing a pathway of communication between separate cortical regions and constituting a relay that facilitates transmission of subcortical information to the cortex [19]. The cytoarchitectonic research made by Brodmann [20] proposed the uniqueness of prefrontal cortex in primate species, and more recent evidence indicated specific functional characteristics presumably present in all mammalian species [21].

**Figure 1:**
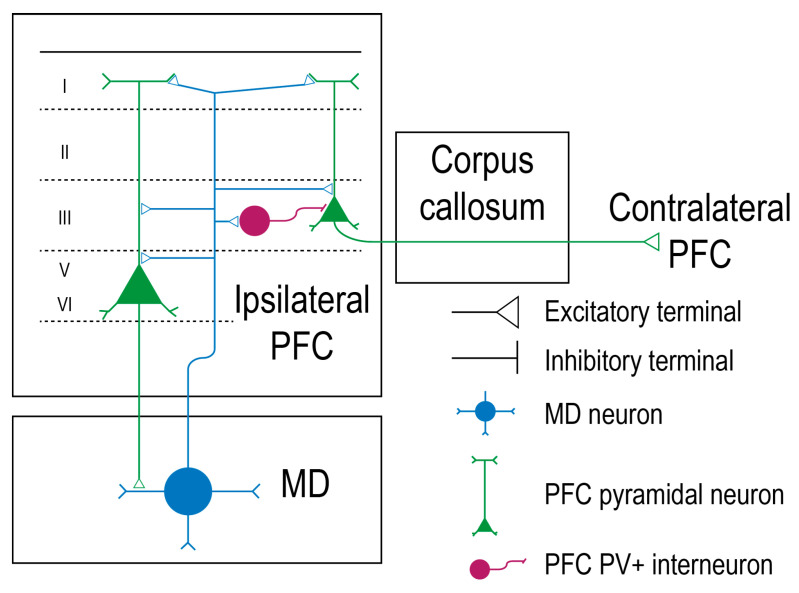
Mediodorsal thalamic neurons have bidirectional connections with prefrontal cortex pyramidal neurons. The pyramidal neurons of the prefrontal cortex (PFC), represented in green, distribute their soma in layer 3, 5, and 6. The deep layer pyramidal neurons project back to the mediodorsal thalamus (MD), forming a loop. The mediodorsal thalamocortical neuron is represented in blue and synapses with the pyramidal neurons. The parvalbumine-expressing inhibitory interneurons (PFC-PV+) are represented in dark purple, while the excitatory connections are represented as a triangle [24].

In humans and in rhesus monkeys, MD is situated in the middle third of the thalamus, between the internal medullary laminae and the periventricular gray matter [22]. MD is organized in a specific way so that each of its subdivisions is bidirectionally linked with different prefrontal cortical regions. MD receives robust input from cortical layers 5 and 6 and projects back to the cerebral cortex. In this way, cortico-thalamo-cortical circuits are being created [9,23]. In rat, the prefrontal cortico-thalamo axon terminals have round vesicles that synapse with the distal parts of the MD dendrites. The thalamo-cortical projections to PFC also own synaptic round boutons and synapse with the dendritic spines of the basal or apical dendrites of the pyramidal neurons. Those prefrontal pyramidal neurons present their soma in cortical layers 3, 5, and 6. Interestingly, few pyramidal neurons belonging to layer 3 are callosal, and only deep-layer pyramidal neurons form monosynaptic loops with the MD. This fact indicates that the MD can activate ipsilateral layers 3, 5, and 6 in the PFC, but only neurons in layers 5 and 6 can send back the information to the MD that stimulated them. Layer 3 pyramidal neurons that were activated by the MD will further project to the contralateral PFC. Both mediodorsal-prefrontal and prefrontal-mediodorsal pathways are glutamatergic and excitatory (Figure 1) [24]. The neuronal activity of the thalamus is greatly coherent with the cortical one, but the cortex exercises a higher control over the thalamus in all species, given the numerous corticothalamic inputs and the common synaptic organization of the thalamic relay neurons [25]. The existence of a top-down regulation facilitates behavioral control and offers the capacity to guide thoughts in a manner that is coherent with inner goals [26]. In non-human primates and in humans, the main efferents of the midline group of the thalamic nuclei are the medial and lateral prefrontal cortices and the orbitofrontal cortex [2].

In humans, the ventral medial prefrontal cortex (vmPFC) participates in the process of regulating several mood disorders accompanied by anxiety. The vmPFC contains two different regions: the prelimbic cortex (PL, situated dorsally) and the infralimbic cortex (IL, situated in the ventral part of the vmPFC). Particular attention should be paid to terminology, as over times different names have been assigned to homologous regions of different species. Researchers proposed that rat IL is homologous to monkey and human Brodmann’s area 25, while rat PL corresponds to Brodmann’s area 32 in monkeys and 32 pl in humans with similar cytoarchitectures. Other studies suggest that rat PL corresponds to human 32 ac and monkey 24 c [27]. While human 32 pl is located in the ventrocaudal genu of the corpus callosum, similar with area 32 in monkeys, both human area 32 ac and monkey area 24 c are found in the dorsoanterior genu of the corpus callosum. They have almost identical cytoarchitecture (similar densities of pyramidal cells) [28].

In monkeys, the caudal part of the frontal pole (area 10) has four main cytoarchitectonic regions known to receive thalamic afferents: areas 14 and 25 (situated in the ventral part) and areas 24 and 32 (found in the dorsal part) [29,30]. Researchers reported that the magnocellular division of MD (MDmc) sends projections to the ventral precallossal and subcallosal areas 14 and 25, as well as to the ventral subcallosal part of area 32. The parvocellular division of MD (MDpc) projects to area 14, dorsal precallosal part of area 32, supracallosal area 24, but also to polar area 10, an insufficiently described region in relationship with the thalamus [31]. Besides, studies showed that the magnocellular division projects more than the parvocellular in area 14 [22,32,33]. Similar to the parvocellular division, the densocellular one projects also to polar aria 10 and supracallosal area 24 [31]. Other studies sustain that the magnocellular is linked preferentially with the orbitofrontal cortex (Brodmann areas (BA) 11, 13, 47/12) and ventromedial prefrontal cortex (vmPFC: areas 14, 25, 11, 13, and 12), a fact that certifies the limbic system appurtenance. It also receives a nonreciprocal input from ventrolateral (vlPFC: area 45) and medial prefrontal cortex (the dorsal anterior cingulate cortex (dACC): area 32 from the ventral and caudal sides). The parvocellular is reciprocally linked with the dorsolateral prefrontal cortex (BA 9, 45, 46) and receives unidirectional input from orbitofrontal cortex (area 12, 13), ventrolateral prefrontal cortex and the dACC (supracallosal area 24 and from the dorsal and rostral aspects of precallosal area 32 and 14). The caudodorsal division has reciprocal connections with the medial prefrontal cortex (BA 14, 24, 32) [6,9,11,34,35,36,37]. Although there are bidirectional connections among separate regions of the MD and specific areas in the prefrontal cortex, those exact cortical regions also receive fibers that originate in other specific thalamic nuclei, such as the anterior ventral complex of the thalamus. In addition, in both rodents and primates, the MD spreads its fibers to the posterior premotor, primary motor, and anterior cingulate cortex, too [21,22,38,39,40]. Even though the PFC is an essential part of the frontal lobe and has vigorous reciprocal connections with the MD, it must be noted that the prefrontal is still a very complex structure and should not be defined only by its anatomic relationship with this particular thalamic nucleus [21].

## 4. Mediodorsal Thalamic Relationship with the Medial Temporal Regions

In monkeys, numerous cortical temporal areas (superior temporal gyrus, temporal pole (area TG), and parahippocampal gyrus (areas TH/TF)) send nerve impulses to numerous medial prefrontal cortical regions. For instance, area 24 receives input from the anterior medial nucleus and medial thalamic nuclei but also from the entorhinal cortex. Areas 25 and 32 receive input from the entorhinal cortex too. Therefore the medial temporal lobe responsible for object recognition memory can transmit information and influence the prefrontal cortex through the MDmc, especially areas 14, 24, 25, and 32 [31]. All areas 24, 25 and 32 surround the corpus callosum’s rostrum. Other studies also sustain the role of MDmc in relationship with the association cortex of the temporal lobes. In primates, the entorhinal (area 28) and perirhinal (area 35) cortices communicate with the MD through the inferior thalamic peduncle and the ventroamygdalofugal pathway. However, in rodent brain, only the perirhinal cortex seems to be linked with the MD [6,36,41,42]. Investigators stated that the MD and the temporal cortex do not have a bilateral relationship in monkeys: solely MD sends projections to the entorhinal cortex and the temporal pole [31,43,44]. Several studies reported intriguing data in monkeys: perirhinal 35, 36 and inferior temporal (TE) areas do not project to the medial prefrontal cortex, but to the ventral and ventrolateral part [31,45,46,47,48,49,50,51]. Moreover, there is little evidence of a reciprocal communication between the perirhinal and anterior cingulate cortices (area 24), which may sustain their relationship role in memory [31,50,52]. Besides, the cingulate cortex is known to have two regions that function differently: the anterior part cooperates with the MDmc, while the posterior part is subordinated to the anterior thalamic complex [2].

The object recognition memory is formed by a complex system, consisting of multiple cortical and subcortical regions collaborating. Researchers consider that in primates, the medial temporal cortex, MDmc, vmPFC, and orbitofrontal cortex are part of the network implicated in this kind of memory. The explanation is that areas 11, 13, 14, and 25 receive indirect input from the MDmc and direct input from the medial temporal cortices. More specifically, the entorhinal, temporal polar, and parahippocampal cortices project directly to the ventral medial areas 14 and 25. In contrast, the inferior temporal and perirhinal cortices send uninterrupted projections to the 11 and 13 regions, which are found in the vicinity of 14 and 25 areas [31,32,36,44,47,53,54].

## 5. Mediodorsal Thalamic Relationship with Subcortical Structures

The thalamus represents an essential structure with a significant role in the expression of the emotional drive and goal-directed behavior [55]. Data indicate tight bonds between the central and basolateral nuclei of the amygdala and the magnocellular MD (in monkeys) [56] or medial MD (in non-primates) [57]. More specifically, in monkeys, fibers emerging from the posterior amygdala project to the antero-medial MD, while the anterior amygdala projects to the postero-medial MD. However, considering the distribution of the MDmc afferents, several researchers divided this area into four different regions, ventro and dorsomedial, ventro, and dorsolateral MDmc, arranged anteroposteriorly in three-dimensional clusters of columns. Studies demonstrated that the fibers emerging from the amygdaloid complex of the monkeys end mostly in the anterior third of MDmc, whereas the amygdalohippocampal region and the parvicellular accessory basal amygdaloid nucleus end in the dorsomedial and dorsolateral quadrants. Moreover, the periamygdaloid cortex, together with the parvicellular basal nucleus, project ventromedially to the MDmc, while the ventrolateral quadrant receives projections from the magnocellular basal nucleus, magnocellular accessory basal nucleus and the lateral nucleus of the monkeys [36,56]. Still, the amygdala projects less to the MD than it does to the striatum and prefrontal cortex [16]. The amygdaloid cortex sends massive projections to the medial and orbital prefrontal cortex (monkey areas 12, 14, 24, 25, 32) and sparse ones to the dorsolateral prefrontal cortex of the rat, cat, and monkey [58,59]. Besides, the medial part of the mediodorsal thalamus sends back collateral fibers to the basal amygdaloid complex and anterior cortical nuclei of the amygdala. At the same time, the lateral sides of MD and the central MDpc do not communicate straightforwardly with the amygdala and medial temporal lobes [6].

Data indicate that MDmc can represent a potential multisensory relay for prefrontal cortex. Sensory association areas send fibers to different structures, such as the amygdaloid complex and the entorhinal and the temporal polar cortices through cortico-amygdaloid and cortico-cortical circuits, indicating that the information provided to the MD has an essential contribution to the cognitive processing of certain stimuli, primarily visual (in monkeys) and olfactory ones (in monkeys and hamsters) [60,61]. The olfactory system seems to be very well connected to the MD of monkeys, as axonal tracing revealed direct connections between MD and the deep piriform cortex and several olfactory bulb related structures, such as the olfactory tubercule, the anterior cortical amygdaloid nucleus and the periamygdaloid and the anterior entorhinal cortices [36,62]. Moreover, the uniqueness of the MD thalamic nucleus lies in the ability to receive information from the primary olfactory cortex and to send it afterwards to the orbitofrontal and insular cortices, favoring associative olfactory functions. Another part of the MD acts as a sensory relay for pain, as it receives afferents from the lateral spinothalamic and trigeminothalamic tracts, passing the information subsequently to the frontal lobe.

Thalamus is known to have a fundamental role in performing a variety of motor tasks, as it is connected with the brainstem structures and basal ganglia. The parvocellular division of the mediodorsal thalamus receives input from the internal segment of the globus pallidus, locus ceruleus, reticular formation, ventral tegmental area, and the reticular part of the substantia nigra from the midbrain (last two projections: non-dopaminergic). Similarly, the lateral part of the mediodorsal thalamus (which comprises the densocellular and pars multiforms) gets input from the internal segment of the globus pallidus, locus ceruleus, reticular formation, as well as a non-dopaminergic input from the ventral tegmental area and the reticular part of the substantia nigra. However, it receives afferents also from the median raphe. Moreover, the lateral MD project back to the basal ganglia. The magnocellular MD receives input from the ventral pallidum, locus ceruleus, reticular formation, dorsal raphe, ventral tegmental area, and also the reticular part of the substantia nigra (dopaminergic) and basal forebrain (GABAergic) [6,55,63,64,65,66,67,68]. Researchers consider the striatum as the input of the basal ganglia, while the internal pallidum, the ventral pallidum, and the reticular part of substantia nigra are regarded as their output. Moreover, multiple parallel circuits have been discovered between the striatum, basal ganglia, thalamus, and prefrontal cortex in both rats and monkeys, noting the strong link between the thalamus and the striatum [21,55,69,70]. MD seems to be part of three segregated neural cortical-subcortical circuits in rats or in primates. The medial circuit involves bidirectional connections between the MDmc and the orbitofrontal and ventromedial prefrontal cortices, as well as some neural afferents from the ventrolateral prefrontal and rhinal cortices, amygdala, ventral striatum, and ventral pallidum. The central circuit connects the MDpc bidirectionally with several structures, such as the dorsolateral prefrontal cortex and area 10, and brings afferents from the orbitofrontal cortex, dorsal anterior cingulate cortex, globus pallidus, and dorsal striatum. The lateral circuit links the intralaminar nuclei with globus pallidum, dorsal striatum, prefrontal cortex, and the frontal eye fields [6]. Studies claim that the mediodorsal thalamus receives projections from both cerebellum and basal ganglia. Deep cerebellar nuclei have axonal terminations on the entire surface of the thalamus in human and non-human primates, whereas basal ganglia project on restricted areas from the MD: in parvocellular, densocellular and pars multiforms regions [71,72,73].

There is evidence that the basal ganglia and the cerebellum interact with the human neocortex, especially with the cortical motor areas [74], through different functional projection systems that are interconnected in the thalamus. This relay receives most of its projections from the cerebellar dentate nucleus whose fibers pass anteriorly or through the red nucleus [75] but also from the basal ganglia, which projects to the thalamus via globus pallidus. Both networks get into the ventral part of the thalamus and form the pallido-thalamic and cerebello-thalamic fascicles [76]. In humans, magnetic resonance imaging and tractography studies unveil a decreasing gradient for pallido-thalamic connections, oriented antero-posteriorly, in several thalamic regions, including midline, as well as a decreasing postero-anterior gradient for the dento-thalamic connections in several thalamic regions, including the medioventral and lateral mediodorsal nucleus. Moreover, both kind of projections were hemispherically lateralized to the left thalamus.

Regarding the dentho-thalamic connectivity, the lateral parts of the parvocellular are highly connected with the medial division of the dorsal thalamus and medioventral nucleus, but the magnocellular has a low degree of connectivity in the right hemisphere. The pallido-thalamic connectivity is high between the inferior parts of parvocellular, lateral parts of magnocellular, and the medioventral nucleus. However, the pallido-thalamic projections can be found more medially and anteriorly than the dentate-thalamic projections, with an essential overlap in the intralaminar nuclei and midline regions in mice [77].

## 6. Mediodorsal Thalamic Relationship with the Cerebellum

The fastigial nucleus is phylogenetically the oldest in the cerebellum. It forms crucial connections with motor and non-motor systems, thus being implicated in various emotional activities and contributing to a coordinated response to internal and external stimuli [78]. The fastigial glutamatergic projection neurons can modulate the cortical circuits by crossing midline and synapsing with the midbrain and thalamus [79]. Disruptions of the cerebello-thalamo-cortical circuits seem to occur in several psychiatric disorders. Emerging resting-state functional connectivity studies showed a severe decrease in the mediodorsal thalamus-cerebellar connectivity in schizophrenia and mild reductions in the MD connectivity in bipolar disorder [80].

**Figure 2:**
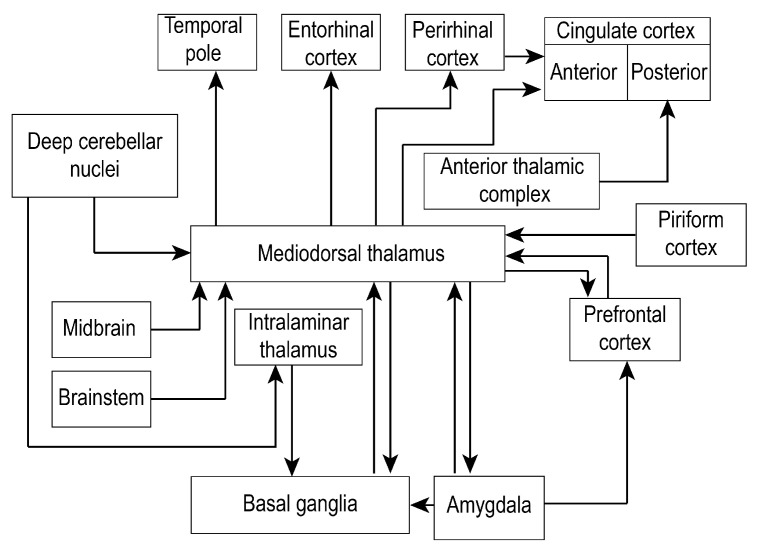
Mediodorsal thalamus establishes numerous connections with the prefrontal cortex, medial temporal regions, subcortical regions, and cerebellum. Due to the highly complex connectivity of the mediodorsal thalamus to other brain regions, only the most relevant connections (black arrows) are shown, making this a non-exhaustive representation.

Interestingly, recent research proposes the use of optogenetics in the treatment of schizophrenia: deep cerebellar nuclei have necessary interconnections with the prefrontal cortex. Therefore their stimulation is believed to improve cognition in humans [81]. The disynaptic connection between the dentate nuclei and the anterior cingulate cortex is essential for attention, working memory or other cognitive functions related to the mediodorsal thalamus. In addition, several impairments such as visual, linguistic, and executive dysregulations can occur in cerebellar lesions. The bidirectional connections between the cerebellum and the cerebral cortex is fundamental for emotional regulation also. This function is possible in rodents and humans due to existing projections that originate in the fastigial nuclei, reaching multiple limbic system structures [82,83]. The fastigial nucleus was even considered part of the limbic cerebellum and an extension of the Papez circuit [84,85]. Researchers suggest a direct connection between the fastigial nucleus and the thalamus: in cats, fastigial electrolytic lesions produced the degeneration of the fibers projecting into and through the midline thalamus, towards the medial forebrain [8]. It projects, therefore, to frontal cortical regions associated with behavior, forming cerebello-cortical loops via the thalamus, including via medial dorsal nucleus [16]. In the rat, it was demonstrated that simulated lesions (obtained by deep brain stimulations) of the MD could increase impulsive behavior without altering the motor function. The experiment decreased the expression of c-Fos (an immediate early gene encoding a transcription factor that influences the cell cycle, proliferation and differentiation of cells, as a response to a stimulus [86]) in deep cerebellar nuclei and increased it in the prefrontal cortex. This phenomenon reduced the selective attention by disrupting the cerebello-thalamo-cortical pathway [87]. Cognitive impairment can also result when changing the neural activity in the dentate nucleus. Its glutamatergic projections to contralateral MD can alter the dopamine balance in the rat mPFC [88]. Cerebellar involvement in emotions like fear has recently been proven: The vermis inactivates during the consolidation of the memory. Moreover, both vermis and cerebellar cortex participate in forming new associations during the acquisition phase of fear conditioning, and the occurred plastic changes affect both excitatory and inhibitory synapses [89]. All these findings demonstrate the cerebellar capacity to modify the thalamic activity and influence multiple functions in which MD participates (Figure 2).

## 7. Mediodorsal Thalamus Cytoarchitecture

**Figure 3:**
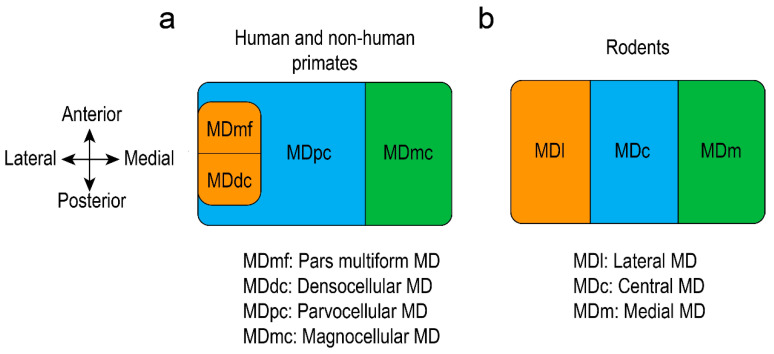
Graphical representation of the mediodorsal thalamus (MD) in human and non-human primates (**a**) and rodents (**b**).

Rodents, humans, and non-human primates present a similar mediodorsal thalamic cellularity [6,31,90]. MD is the largest nucleus in the medial thalamus and can be divided into several areas, depending on species. In rats, the MD is composed of a medial part (MDm), a central part (MDc) and a lateral part (MDl) [17,57], while some researchers mention also the existence of a paralamellar area, situated at the lateral margins of the MD [91]. The anatomy and functional connectivity of the MD helped to reveal the corresponding subdivisions in non-human primates. Studies showed that the magnocellular division (MDmc) of the monkey, situated in the antero-medial MD, corresponds to the medial MD of the rat. The parvocellular division (MDpc) of the monkey MD, situated in the centro-lateral part, corresponds to the central MD in rats, whereas the lateral part of the mice MD corresponds to the densocellular (MDdc, situated in the latero-caudal part), and pars multiforms (MDmf, which can be found in the latero-rostral part) of the monkey MD [6,7,32]. Other researchers propose different parcellation in monkeys: The magnocellular MD represents the medial part and the parvocellular MD the lateral part [9]. The MDmc was further divided into a paramedian and a fibrous segment, while sometimes a poorly myelinated segment was described caudodorsally in monkeys [33]. Others considered that MDmc of monkeys corresponds to medial and central parts of rat MD, while MDpc, MDdc, and MDmf of monkeys correspond to the lateral subdivision of the MD in rats [24]. The human MD is substantially bigger that the non-human primates one [6] and can be segregated also depending on the connectivity with the prefrontal cortex, which is similar to the non-human primates. Human MD is composed of a magnocellular division (situated medially), an adjacent parvocellular division, and a lateral division [2]. The MDpc sits lateral to the MDmc and extends towards the most caudal portions of the MD, while the MDmf (called often pars paralamellaris) is located in the most lateral part of the nucleus and MDdc in the caudal part, lateral to the MDpc and habenula [22] (Figure 3). Other histological studies showed that the human MD parcellation can be correlated with the non-human primates, revealing the MDmc division in the medial side, MDmf in the ventral side, and MDpc in the dorsolateral side [13]. The divisions are distinguishable when using acetylcholinesterase staining. Studies have proven that the anterior parts of the mediodorsal thalamus are not as powerfully stained as the posterior parts. Moreover, the posterolateral part (adjacent to the internal medullary lamina) is the heaviest stained, but irregular patches of lighter and denser staining are visible throughout it [16].

This exact disposition and lack of homogeneity have also been seen in human brains when using acetylcholinesterase. The human mediodorsal nucleus was seen as a large structure, which was covered by the internal medullary lamina in the anterior, posterior, lateral, and ventral sides. Here, the anterior parts appeared lighter than posterior ones, while the postero-lateral parts appeared the highest stained. Besides, Nissl staining revealed a similar pattern of distribution and inhomogeneity too. The cell population are grouped in a medial area composed of large, dark stained neurons, representing the magnocellular region. Mixed sizes and staining intensities were visible for the ventral region, pars multiform, whereas the parvocellular region showed smaller variably stained cells [13].

The overall aspect of human MD cell populations corresponded to the one observed in monkeys [16]. In rats, Golgi technique could evidence the distribution and dendritic domain of the neurons, which again proved the division of the mediodorsal thalamus into several recognizable segments. The technique revealed two types of relay neurons in the MD whose shapes, dimensions, and dendritic arborization allowed their classification and naming as stellate and fusiform neurons.

Interestingly, both types distribute their dendrites in the immediate vicinity of the cellular body and do not exceed the subdivision of which they are part. The majority of the neurons are stellate, large (2531–µm by 1823–µm), have an elliptical cell body shape, and spherical dendritic field, with dendrites that emerge radially in all directions and can divide into secondary, tertiary or higher-order branches that spread up to 30µm away from the cellular body. This type of neurons can be found in the core of each mediodorsal subdivision, but they are located especially in the central MD. The fusiform neurons are rare and have a small round-shaped cell body (711–µm diameter, reaching up to 29 µm in total length), with an elongated (bipolar), sparse dendritic field, usually oriented dorso-ventrally. Fusiform neurons spread their dendrites parallel to the segmental borders while being located in their closeness. They are present in all segments but very rarely can be seen in the central MD. The neurons appear therefore in a great major in the ventrolateral part of the mediodorsal thalamus.

Nonetheless, it is not always easy to distinguish the neurons because an intermediate dendritic pattern can occur in the MD [24,90,92] Some researchers proposed a high similarity in the cellular morphology and dendritic arborization throughout the MD [93]. This arrangement promotes a coherent activity and a fast spread of information in the relay neurons, even though a minimal number of GABAergic interneurons may connect them.

## 8. Mediodorsal Thalamus Electrophysiological Responses in Relationship with Limbic-Related Areas

An essential characteristic of the MD neurons is that they can fire in either a tonic or bursting mode, a fact that influences the level of consciousness. The thalamic afferents and the cortical feedback can exert a powerful effect and modify the membrane potential of the MD, which is in fact responsible for the dual firing mode [94]. When the membrane potential is over −55 mV, the administration of a brief depolarizing current will provoke repetitive fast spikes, but if the membrane potential is less than −60 mV, the same stimulus will trigger bursts, followed by a 150,180–ms refractory period. This feature allows the thalamus to act differently, depending on the state of cortical arousal: In sleep, the reticular nucleus exerts its powerful influence on the neurons, hyperpolarizing them. The activity can be recorded on the EEG as high-amplitude slow waves of spindles and delta frequencies. In this way, thalamic neurons can function as a very efficient relay for the transmission of the sensory information from the nonspecific brain stem afferents to the specific cortical areas. The thalamic neurons can pass on the information from the inferior to the superior centers in a short period when operating in the tonic mode. During tonic firing, those neurons can group and fire synchronously at approximately 40 Hz. This frequency facilitates sensory transmission through the relay and represents the value at which they function best. Usually, during wakefulness, the oscillatory activity is maintained in the gamma range by the cortico-thalamic feedback, offering thus a coherent transfer of information between cortex and thalamus [93].

Studies mention the influence of dopamine on the mediodorsal excitability in rat. The quinpirole (D2 receptor agonist) administration triggered a 5.9 ± 4.9 mV hyperpolarization of the resting membrane in 51.3% of the tested neurons. Only 19% of the neurons suffered a 3.4 ± 0.7 mV membrane depolarization. Other exciting effects were reported after the quinpirole administration: The evoked spikes were followed by spike after hyperpolarization, characterized by larger amplitudes and increased duration. Dopamine enhanced, therefore, the excitability of the membrane and facilitated the apparition of low-threshold spikes (210–spikes/discharge), which compose the rhythmic burst firing described in the thalamic neurons [95]. However, arrhythmic bursts have also been observed in animals and humans. They are believed to be pathological, as all humans who manifested them had a neurological disorder [96,97].

Many of the limbic structures influence the prefrontal cortex by changing the thalamic activity. The neural networks connecting all these areas control several executive functions. One of them is the working memory, which requires the storage of a trial-unique information in order to guide future behavior after cognitive processing. In addition, goal-directed behavior and behavioral flexibility were proven to depend on the MD but mainly on the prefrontal activity. Those functions involve the ability to adapt to changes by making new stimulus–outcome or response–outcome associations [98]. Recordings in freely-moving mice showed an increased MD-PFC theta- (4–12 Hz) and beta-range (13–20 Hz) synchrony during acquisition and execution of a working memory assignment, while the mediodorsal individual neuronal spikes synchronized with the beta oscillations of the prefrontal local field potentials during the choice phase of the task [99].

Moreover, it seems that task performance and working memory improve when the excitability of the MD neurons increases [100]. Lidocaine injections in the MD and contralateral PFC and injections in the accumbens and PFC disconnected the implicated regions and disrupted the performance on a delayed task. However, the interruption of the MD-accumbens path did not have a definite effect, suggesting a serial flow of information in the brain [101]. Moreover, the electrical single pulse stimulation of the nucleus accumbens generated mainly a decrease of the firing rate in the mediodorsal thalamus (13.5 ± 1.9 ms mean latency) and a long duration of neuronal inhibition. However, in 16.6% of the neurons, the extracellular recordings also revealed an excitatory response followed by an inhibitory period with a 3.5 ± 1.2 ms latency, demonstrating the modulatory role of nucleus accumbens over the mediodorsal thalamic activity and thalamic dependency on motivational stimuli [102].

The hippocampal-prefrontal circuits can also intervene in the working memory. The MD and the ventral tegmental area projections connect with the hippocampal-prefrontal pathway and modulate its functions. Extracellular single-unit recordings in rats revealed a monosynaptic orthodromic spike in the medial prefrontal areas (prelimbic cortex) after the fimbria/fornix (FF) pulse stimulation, with a mean evoked firing latency of 11 ± 0.8 ms (range 7–17 ms). The results also showed that 9.2% of the prefrontal neurons studied had an antidromic response to MD—mean spike latency 11.2 ± 0.9 ms, range 7—18 ms, while 24.6% of the PFC neurons determined an antidromic spike back to the ventral tegmental area (VTA)—mean latency 9.6 ± 1.0 ms, range 4–19 ms. Interestingly, 6.7% of PFC neurons seem to project back to both VTA and MD.

Interestingly, 12.4% of the rat hippocampal efferents overlapped with the MD orthodromic input in mPFC. In this case, the mean spike latency after MD stimulation was 7.6 ± 0.6 ms. Nevertheless, a particular differentiation of the hippocampal-PFC projections can be done depending on the final projecting areas: Neurons that projected to the MD were found in the rostral parts of the PFC (infralimbic and prelimbic cortices). In contrast, neurons that projected to VTA were found in the dorsal prelimbic cortex. When a conditioning pulse was administered in the FF with 10–500 ms before applying a test pulse in MD, a firing rate depression occurred in the PFC and lasted for approx. 100 ms. The same pattern was observed if the MD was stimulated before FF.

Burst stimulations of the MD revealed two populations of PFC neurons that respond to hippocampal stimulation in rats. One major population (66% of studied neurons) was inhibited for 250 ms after the MD burst, while the other showed an increase in the hippocampal-evoked firing rate. The tetanic stimulation of the MD produced a short-term (5 min) potentiation in the hippocampal–prefrontal firing, suggesting the modulatory role of the MD over the hippocampus and an increased capacity of PFC neurons to respond to the hippocampal activity [103]. Some researchers sustain that, even though both PFC-MD and MD-PFC pathways are glutamatergic and excitatory, the mediodorsal thalamic axons synapse with the PFC GABAergic interneurons [24] and trigger a three times higher excitatory postsynaptic potential amplitude on the fast-spiking interneurons than it does on the regular-spiking pyramidal neurons. The excitatory effect obtained after the MD stimulation is followed by an inhibitory postsynaptic potential [104].

Researchers sustain that the optogenetic stimulation of the MD neurons induces the activation of layer I, III, and V of the dorsal anterior cingulate cortex, but the neurons found in layer III seem to receive the majority of the afferents. Photostimulation of the MD neurons in mice triggered excitatory polysynaptic currents in the third layer pyramidal neurons after approximately 5.48 ms. Usually, inhibitory postsynaptic currents occurred at approximately 10.24 ms. This inhibition turned out to happen mainly because of the parvalbumine-expressing interneurons, which can be activated by the MD projections to the dorsal anterior cingulate cortex. Since parvalbumine-expressing interneurons are fast-spiking (1.22 ± 0.14 ms), it was suggested that the MD creates a disynaptic inhibition and controls the pyramidal neuronal activity by using the characteristics of this type of interneurons [100].

## 9. Mediodorsal Thalamus Neuromodulation

The thalamic nuclei form a vital system that can receive and forward the information in the brain, including the neocortex, the basal ganglia, or other limbic related structures. The majority of the thalamic synapses are modulatory, connected with higher-order cortical areas. The medial dorsal thalamic nuclei belong to the higher-order thalamus. They can connect cortical and subcortical brain regions, maintain the synchrony among distinct areas, and direct the wide spreading of the information. All those qualities are believed to favor cognitive and emotional processing, as several impairments have been described after MD lesions. Attention and memory deficits, learning difficulties and emotional instability appeared in human MD damage [105,106]. Thalamus presents extensive reciprocal connections with the cerebral cortex and acts like a principal regulator of the functional cortical connectivity, strongly influencing cognitive functions. It was demonstrated that MD is able to show a temporal tuning of two conflictual attentional targets but cannot distinguish between the task rules of selection. The bidirectional optogenetic manipulations during this type of task revealed that MD inputs enhance the lateral connectivity between PFC neurons but cannot dictate the baseline excitability and the direct, absolute tuning. These findings suggest that the cognitive concepts of attention and decision-making are directed by a set of algorithms that vary according to behavioral demands and distribute across cortical regions when engaged in cognitive tasks. This indicates the existence of a functional connectivity between thalamus and distinct clusters of cortical neurons [107]. Taking all of the above into consideration, it was suggested that the communication between the mediodorsal thalamus and the cortex is fundamental for fear extinction also, since attention is needed to decrease the unpleasant thoughts and the intensity of the feelings [108].

Thalamic modulators use different neurotransmitters and influence various executive functions [109]. The most critical input of the thalamus may be represented by the glutamatergic pyramidal cells from the cortical layer VI. Almost 50% of these neurons act as a center of integration because they receive afferents directly from the thalamus and all cortical layers simultaneously. The thalamostriatal endings can be modulated by histamine via H3 and H4 presynaptic receptors. Hypothalamic tuberomammilary histaminergic neurons project to the thalamus and only exert their action during wakefulness, which is directly proportional to the neuronal degree of activation. Therefore, histamine can regulate the attention and the sleep-wake cycle. Another thalamic neuromodulator is choline. Like histamine, choline is involved in regulating vigilance, attentional levels, and sleep-wake cycle but can do this by controlling the brainstem input to the neocortex through the thalamus. The mediodorsal thalamus receives cholinergic fibers from the laterodorsal tegmental nucleus and the intralaminar nuclei. Serotonin also profoundly influences the sleep-wake cycle by varying the firing mode in the intralaminar, anterior, and midline thalamic nuclei. This neurotransmitter has a crucial role in maintaining the mood balance: Low serotonin levels can induce emotional disorders and depression and alter social behavior. The serotoninergic afferents come from the medial and lateral midbrain. Afferent noradrenergic fibers of the thalamus originate in locus coeruleus and have a fundamental role in the motor system [2].

During sleep, neurons are hyperpolarized, in a continuous inhibition phase, thus, the communication between different structures is slowed down. However, whenever a new stimulus is presented, the cortex can be alerted by changing the thalamic firing pattern from the burst to the tonic mode. Thalamic neurons present, therefore, two sorts of physiological responses: tonic and burst firing. The tonic firing of singular action potentials occurs when the membrane is depolarized, whereas bursts of high-frequency action potentials occur when the membrane is hyperpolarized [94,110]. These thalamic firing patterns have numerous physiological contributions, and MD seems to play an essential role in the emotional processing, giving its dense interconnections with the medial prefrontal cortex: Tonic firing facilitates the extinction, while bursts suppress it [111]. The hyperpolarization activates the T-type Ca^2+^ channels, which are responsible for the burst firing. Shifting between the burst mode to the tonic one is possible due to the metabotropic glutamate receptor type 1 which depolarizes the thalamocortical neurons when they are stimulated by the glutamatergic cortico-thalamic inputs [112]. Researchers suggest that this phenomenon is feasible on the strength of the thalamic relay neurons with bushy dendritic symmetrical fields in all nuclei and all species, except the cat.

Moreover, the electrical properties of all the glutamatergic MD neurons are believed to be similar, given the characteristics of the membrane, synapses, and circuitry. This implies an identical pattern of response to electrical stimulation. Moreover, a robust inhibitory effect is exerted by the reticular nucleus via GABAergic efferences to the MD neurons, which influences the intrinsic circuitry [93]. The four subdivisions of the MD have connections with separate cortical regions, but the containing relay neurons can unite and combine the actions of different cortical areas that are influenced solely by one thalamic nucleus [6,113]. The MD thalamic neurons can be either parvalbumine-positive or calbindin-positive, which spread into the cortex through a vast thalamic matrix. However, the transmission can still be influenced by the GABAergic and peptidergic inhibitory interneurons found in all thalamic nuclei. Besides, the electrical synapses found in the reticular nucleus can participate in the inhibition of the thalamocortical projections. These electric synapses exercise a high control over the projections and allow an exact synchronization of the existing circuits [2].

## 10. Conclusions

The evidence presented here illustrates the complexity of the MD and the essential role in the entire brain activity. Through the multitude of anatomical connections with cortical and subcortical regions, the MD can receive, integrate, and direct valuable information to essential areas related to cognition and emotion. Moreover, the cytoarchitecture of the structure allows MD to change both the intrinsic and extrinsic neural activity and influence numerous functions operated by the brain. However, further research should be advanced to better understand how MD cooperates with PFC, limbic system, and other subcortical regions, in order to integrate cognitive and emotional processes for proper adjustments to the environment.
